# Climate Anxiety in Perspective: A Look at Dominant Stressors in Youth Mental Health and Sleep

**DOI:** 10.1111/nyas.70057

**Published:** 2025-09-15

**Authors:** Charles A. Ogunbode, Lois Player, Su Lu, Miriam Sang‐Ah Park, Rouven Doran

**Affiliations:** ^1^ School of Psychology University of Nottingham Nottingham UK; ^2^ Department of Psychology University of Bath Bath UK; ^3^ School of Applied Social Sciences De Montfort University Leicester UK; ^4^ School of Social Sciences Nottingham Trent University Nottingham UK; ^5^ Department of Psychosocial Science University of Bergen Bergen Norway

**Keywords:** climate anxiety, eco‐anxiety, mental health, sleep, wellbeing, young adults

## Abstract

There is growing evidence that climate anxiety is associated with significant effects on the mental health and wellbeing of young people. However, the relative importance of climate anxiety for young people's mental health has hitherto been unclear, as climate anxiety has largely been studied in isolation from other common stressors. This study sought to contextualize the significance of climate anxiety for the mental health of UK young adults relative to other concurrent psychological stressors. We surveyed university students (*N* = 461) and a general population sample aged 18–25 (*N* = 400). The results showed that while climate anxiety was significantly associated with poorer mental health and worse insomnia when examined alone, this association became nonsignificant or greatly diminished when other stressors were considered. Loneliness was found to be the most important predictor of mental health, and financial anxiety the most important predictor of insomnia severity. The findings suggest that climate anxiety, while concerning, may not be an especially dominant factor in young people's mental health. Our research highlights the need to consider the broader context of young people's lives, and the complex interplay of various psychological stressors, in efforts to map pathways between climate change and mental health.

## 1 Introduction

Anthropogenic greenhouse gas emissions are warming the Earth at a rate of 0.2°C every decade [1]. This increase in global temperature is associated with rising sea levels, greater frequency of severe weather, accelerating degradation of the natural environment, and a host of attendant risks to human health and prosperity. The fingerprint of climate change is now detectable in any single day of globally observed temperature and moisture [2]. Climate change impacts are also readily apparent to the public, leading to a rise in the number of people experiencing distress about the climate crisis [3, 4].

Evidence of how direct exposure to climatic extremes affects mental health is well‐established. For example, depression and post‐traumatic stress disorder are more prevalent among communities affected by hurricanes, flooding, and wildfires [5, 6, 7]. Repeated exposure to natural disasters is associated with subsequent worsening of mental health outcomes [8]. Longer‐term gradual environmental changes like rising temperatures and drought have also been linked to suicides and psychological stress [9, 10, 11]. In contrast, there is comparatively little evidence on how mental health and wellbeing relate to people's psychological engagement with the climate crisis. Here, the term psychological engagement refers to people's climate‐related beliefs, emotions, and action tendencies [12].

Young people will live to experience more severe impacts from climate change [13]. They also have less say in, and control over, societal responses to the issue. Accordingly, young people exhibit greater levels of concern about climate change than older generations [14, 15]. A burgeoning literature documents how feelings of anxiety and distress about the unfolding climate crisis are taking a toll on the mental health and emotional wellbeing of young people around the world [4, 16, 17, 18].

Yet, young adulthood is a transitional life stage that is typically characterized by changes, uncertainty, and upheaval [19]. Alongside socioecological insecurities, young people face numerous stressors that they may have inadequate resources to mitigate [16]. Making the transition to a stable and secure adulthood requires appropriate support [20]. In turn, determining what resources or support are necessary or appropriate requires a holistic view of the myriad stressors and risk factors that shape the mental health and wellbeing of young adults. Therefore, the current study aimed to assess how climate anxiety relates to mental health among UK young adults in the broader context of other concurrent psychological stressors.

### 1.1 What Is Climate Anxiety?

Climate anxiety refers to a range of negative emotions that people experience in response to the climate crisis. Eco‐anxiety, a broader term often used interchangeably with climate anxiety, is defined as a chronic fear of impending ecological catastrophe [21]. The Climate Psychology Alliance describes eco‐anxiety as a state of heightened emotional, mental, or somatic distress in response to (perceived) dangerous changes in the climate system [22]. Climate anxiety is not a mental disorder, but it can be considered clinically significant when people's negative emotions become difficult to control [3].

Climate anxiety has been linked to poorer wellbeing and impaired psychological functioning [18, 23]. A longitudinal study conducted in New Zealand found that heightened concern about climate change predicted a residual increase in psychological distress over a period of 1 year [24]. Another study using longitudinal survey data from 11 European countries also identified a significant association between climate change worry and increased risk of anxiety [25]. Climate anxiety is reported to be especially prevalent among young people [23, 26]. In a study of young adults aged 16−25 across 10 countries, most respondents (75%) reported feeling frightened by the future, and a large minority (45%) reported that their worries about climate change negatively affect their day‐to‐day functioning [16]. A subsequent US study involving 15,793 respondents aged 16−25 also found that 43% of respondents believe that climate change is impacting their mental health, and 38% believe that their feelings about climate change are having a negative impact on their daily functioning [4]. Other studies have linked climate anxiety with psychological distress, generalized anxiety, and depression [18, 27, 28]. Scholars have proposed that climate anxiety may be triggered by: (1) grief about loss of places, knowledge, and traditions due to climate change; (2) fear about the potential scope of climate impacts; and (3) uncertainty about the location, timing, and effects of climate change [29].

### 1.2 Climate Change and Mental Health in the UK

The UK faces significant risks from climate change. According to the third UK Climate Change Risk Assessment (CCRA3) report, the likelihood of warmer and wetter winters, as well as hotter and drier summers, in the UK has significantly increased [30]. It is predicted that extreme temperatures and rainfall will also become more frequent, and that sea levels will continue to rise around the country [31]. Flooding is one of the impacts on UK communities expected to result from climate change [32]. Over the past few decades, different parts of the country have been affected by severe flooding. In the winter of 2013/2014, widespread flooding caused severe damage to over 10,000 properties in England and resulted in economic losses totaling approximately £1.3 billion [33].

A year after the 2013/2014 flooding, researchers observed greater prevalence of anxiety and depression among victims of the flood compared with people who were not affected [34]. People who were displaced or evacuated due to the flooding had especially high odds of showing signs of psychological morbidity [35]. Importantly, exposure to extreme events like flooding can also make people become more engaged with climate change [36]. Climate risk perception and willingness to engage in climate change mitigation were significantly greater among victims of the 2013/2014 UK winter floods who subjectively attributed the flooding to climate change [37, 38].

Flooding is not the only issue linked with climate change that poses a mental health risk in the UK. High temperatures have also been identified as a significant health challenge by the UK Climate Change Risk Assessment [38]. Above 18°C, each 1°C increase in temperature has been associated with a 4–5% rise in suicide counts in England and Wales [39]. Patients with mental conditions like psychosis, dementia, and substance misuse also show an increased relative mortality risk (5%) with each 1°C rise in average annual temperatures [40]. Research shows that a 1°C increase in average temperatures over a period of 5 years was associated with a 2% rise in the prevalence of mental health issues among US residents [11].

Furthermore, there is strong media coverage of severe weather and climate change in the UK. This means that people who are not directly exposed to extreme events are still able to witness the suffering and loss wrought on others by climate change. Between 2004 and 2020, the Media and Climate Change Observatory (MeCCO) estimates that six leading UK outlets (The Guardian, Mail, Telegraph, Mirror, Times, and Sun) published nearly 11,000 climate change‐related articles; putting the UK in second place for climate media coverage among 54 countries tracked over the same period [41]. Indirect mediated experiences of climate change, and the negative emotional responses they evoke, may operate as a conduit for climate change impacts on mental health [42]. In previous research with UK young adults aged 16−24, participants reported being distressed by exposure to media coverage of the effects of climate change [17].

### 1.3 Climate Anxiety and Mental Health Among UK Young Adults

It is estimated that one in six adults aged 16 years or older in England meet the criteria for common mental health disorders like anxiety and depression. The proportion of young people aged 17−19 with a probable mental disorder rose from 10.1% in 2017 to 17.4% in 2021 [43]. There is a particularly sharp rise in rates of generalized anxiety symptoms and diagnoses among British young adults [44].

Furthermore, young people in the UK express a great deal of concern about climate change. In a survey of over 2000 young people aged 8−16, three in five respondents reported that they worry about how climate change will affect their lives. A further one in five also reported that they have had bad dreams about climate change [45]. A 2020 report by the Royal College of Psychiatrists revealed that over half (57%) of child and adolescent psychiatrists in England were seeing children and adolescents who are distressed by the climate crisis [46]. Another 2020 study of the UK national population showed age to be a significant predictor of climate anxiety, with younger respondents reporting higher levels of climate anxiety [47].

### 1.4 The Current Study: Hypotheses and Research Questions

#### 1.4.1 How Does Climate Anxiety Relate to Mental Health?

Reyes et al. [18] observed a negative association between climate anxiety and mental health among a sample of Filipino young adults. Chan et al. [48] also found that climate anxiety is associated with depression and generalized anxiety in the United States and China. Additionally, Vercammen et al. [49] observed among UK young adults aged 16−24 that individuals with current mental health diagnoses were more likely to report experiencing moderate to high levels of climate distress. We, therefore, hypothesized that:

**H1**: Climate anxiety is negatively associated with mental health.

We wanted to replicate prior indications of an association between climate anxiety and sleep. Poor sleep is a common symptom of mental ill‐health [50]. Some authors have suggested that sleep is a key explanatory pathway for many negative mental health outcomes arising from climate change [51]. Furthermore, sleep disturbance could be used as a transcultural diagnostic of poor mental health that is less susceptible, than other common self‐report mental health measures, to culturally biased interpretations by diverse research participants [52].

Sleep has a reciprocal relationship with emotion regulation. High emotional arousal can lead to sleep disturbance, which can in turn amplify emotional responses to stressful stimuli [53]. Furthermore, exposure to climate change impacts like high temperatures and severe weather events is associated with sleep disruption and sleep loss [54, 55, 56], and negative emotions mediate the relationship between sleep problems and traumatic exposure to severe weather events [57]. While there has only been a limited exploration of sleep in the climate change literature so far, insomnia is commonly listed among symptoms of climate anxiety [23, 58]. Negative emotions regarding climate change have also previously been linked with insomnia symptoms [52]. Consequently, we hypothesized that:

**H2**: Climate anxiety is positively associated with insomnia severity.

#### 1.4.2 What Is the Relative Importance of Climate Anxiety as a Predictor of Mental Health and Sleep Disturbance Among Young People?

Half of all UK Gen Z^1^ respondents in a 2021 global survey reported that they feel anxious or stressed all, or most, of the time [59]. Climate change was identified as an important concern, although worries about finances, wellbeing, and career prospects were most cited by participants as their main sources of stress. Similarly, in a 2025 poll of UK youth, which combined in‐depth interviews (*N* = 260; age range: 18–29 years) with a representative national survey (*N* = 2307; age range: 16–29), participants identified financial worries, work pressures, and job security or unemployment as the top three issues that made them feel nervous, anxious, or on edge [60]. Climate change and environmental concerns ranked last out of the 13 issues presented to participants in the study.

Reflecting on these findings led us to consider that climate anxiety research has largely overlooked other psychological stressors that may have a concurrent role in shaping young people's mental health alongside ecological concerns. The exception to this trend is a study by Vercammen et al. [49] that explored links between climate distress, mental health, and proenvironmental action among a sample of 539 British young adults.

Vercammen et al. [49] found that climate distress and the frequency of worrying about climate change both showed significant positive associations with young people's frequency of worrying about other issues, including the economy, politics, the COVID‐19 pandemic, and personal relationships. Furthermore, Vercammen et al. developed a relative worry index that was operationalized as the median score for frequency of worrying about climate change (measured with a 5‐point Likert scale ranging from never to very often) divided by the median score of frequency of worrying about seven other key issues assessed (finances, career, COVID‐19, school/studies, relationships, politics, economy). Relative worry index scores greater than 1 reflect a dominance of climate over other worries. Using this formula and the data made publicly available by Vercammen et al. [49], we estimated that 13.8% of participants in their study showed a relative dominance of climate worry. Altogether, the data from this unique study demonstrate that: (1) young people's worries about climate change are not entirely separate from their concerns about other personal and societal issues; and (2) climate worry is not the most salient psychological stressor perceived by many young people. Importantly, the relative salience of climate worry does not equate to its relative importance as a predictor of mental health and wellbeing. It is conceivable that a stressor with relatively strong predictive power for explaining variance in mental health could be masked in perceived salience. Therefore, in this study, we explored two research questions regarding the relative importance of climate anxiety as a predictor of mental health alongside other relevant concurrent psychological stressors:

**RQ1**: Does climate anxiety predict mental health more strongly than health anxiety, financial anxiety, loneliness, and COVID‐19 risk perception?
**RQ2**: Does climate anxiety predict insomnia severity more strongly than health anxiety, financial anxiety, loneliness, and COVID‐19 risk perception?

We identified a set of psychological stressors to evaluate alongside climate anxiety by reviewing the wider literature on young people's mental health. Based on the review, we initially selected financial anxiety, health anxiety, and loneliness. Considering world affairs at the time of designing the research, concerns about the COVID‐19 pandemic and the Russian invasion of Ukraine were also included in the analysis. The importance of the selected stressors for young people's mental health and emotional wellbeing is justified by a range of empirical evidence including research showing that young people aged 16−24 experience the highest levels of loneliness in the UK and globally [61, 62], and that young adults who feel lonely are also more likely to experience symptoms of poor mental health [63]. In the context of the COVID‐19 pandemic, research also revealed that health anxiety, financial anxiety, and anxiety about COVID‐19 were associated with negative mental health outcomes, including depression and anxiety, among young people [64, 65, 66, 67].

The hypotheses and research questions were tested with data from two research participant samples recruited between November 2021 and March 2022. The hypotheses and research questions were preregistered after the start of data collection, but prior to the data being accessed by the research team (https://aspredicted.org/blind.php?x=TLK_ZPX).

## 2 Methods

### 2.1 Participants and Procedure

We gathered data for this study with an online questionnaire administered to two population samples. The first sample comprised diverse university students recruited between November 2021 and January 2022 at three universities in England. Course credit was awarded for participating in the study. The second sample were general UK residents recruited via Prolific, a commercial research panel provider, in March 2022. The purpose of the second sample was to replicate the findings and address a gender imbalance in the student sample.

A target sample size was calculated based on the correlation (*r* = 0.19) previously observed between negative climate‐related emotions and insomnia symptoms among a sample of British research participants [52]. Using G*Power, we estimated that a minimum of 365 participants would be needed to replicate this finding with a two‐tailed test (1 – *β* = 0.95, α = 0.05). We, therefore, aimed to recruit a minimum of 400 participants to accommodate incomplete responses. The eligibility criteria applied in this study were age (18 ≥ M ≤ 25 years) and country of normal residence (UK or constituent nations). Based on these criteria, we excluded the data of 59 participants (out of 520) from analyses of the student sample. In addition, participants who did not disclose their gender (Student sample *n* = 3; General population sample *n* = 2), those who identified as nonbinary (Student sample *n* = 9; General population sample *n* = 13) were excluded from the analyses. The gender‐related exclusions are linked to the fact that we controlled for gender in our analyses based on evidence of binary gender differences in mental health outcomes. Missing data were addressed with listwise deletion.

The final student sample comprised 461 individuals (*M*_Age_ = 19.2 years, *SD*_Age_ = 1.3 years; Women = 86.8%, Men = 10.6%; Ethnicity = 70.1% White/White British). During recruitment of the general UK resident sample, a quota of equal binary gender representation was applied (50% male/female), and an approval rate of 75%−100% on Prolific was required of participants. An effective sample of 400 individuals was achieved (*M*_age_ = 22.1 years, *SD*_Age_ = 2.1 years; Women = 47.5%, Men = 48.8%; Ethnicity = 72.8% White/White British).

The study was advertised as a study of anxiety and mental health. Participants were informed of the focus on climate anxiety when they were debriefed at the end of the study. After full disclosure of the aims of the study, participants were asked to confirm if they wanted to retract their data or were happy for the data to be used for scientific research. The online questionnaire had a mean completion time of 13 min among the student sample and 10 min among the general population sample. Ethical approval for the study was granted by the University of Nottingham School of Psychology Research Ethics Committee (ref: S1372).

### 2.2 Measures

Climate anxiety was measured with the Climate Change Anxiety Scale (CCAS [23]). The scale comprises 13 items that measure two theorized dimensions of climate anxiety: cognitive‐emotional impairment (e.g., “Thinking about climate change makes it difficult for me to sleep”) and functional impairment (e.g., “My concern about climate change makes it hard for me to have fun with my family or friends”). Responses to the items were recorded on a 5‐point Likert scale ranging from “strongly disagree” to “strongly agree.” Prevalence analysis was conducted using an approach adopted from Whitmarsh and colleagues [47], whereby participants were categorized as expressing mild (1.00 ≤ *M* ≥ 2.33), moderate (2.34 ≤ *M* ≥ 3.66), or severe (3.67 ≤ *M* ≥ 5.00) levels of climate anxiety based on their CCAS score. We found that 6.5% and 7.8% of participants in the student and general population samples, respectively, could be categorized as experiencing moderate to severe climate anxiety. Details of the prevalence analysis results, including a breakdown by gender and ethnicity categories, are provided in Table 1.

**TABLE 1:** Prevalence rates of climate anxiety across the student and general population samples.

Sample	Climate anxiety *M* (SD)	Prevalence rates (%) [95% CI]		
*Students*		Mild	Moderate	Severe
All (*N* = 461)	1.40 (0.54)	93.5 [91.2, 96.0]	6.3 [4.1, 8.5]	0.2 [0.0, 0.1]
Female (*N* = 400)	1.41 (0.56)	92.8 [90.2, 95.3]	7.0 [4.5, 9.5]	0.3 [0.0, 0.1]
Male (*N* = 49)	1.24 (0.35)	100.0	0.0	0.0
Asian or Asian British (*N* = 62)	1.59 (0.78)	87.1 [78.5, 95.7]	11.3 [3.2, 19.4]	1.6 [1.6, 4.8]
Black African, Caribbean, or Black British (*N* = 40)	1.35 (0.57)	90.0 [80.2, 99.7]	10.0 [0.3, 19.7]	0.0
Mixed or multiple ethnicities (*N* = 32)	1.36 (0.45)	93.8 [84.5, 100.0]	6.3 [0.3, 15.1]	0.0
White (English, Scottish, Welsh, Irish, or any other White background) (*N* = 323)	1.37 (0.49)	95.0 [92.7, 97.4]	5.0 [2.6, 7.3]	0.0
*General population*				
All (*N* = 400)	1.40 (0.54)	92.3 [89.6, 94.9]	7.5 [4.9, 10.1]	0.3 [0.2, 0.7]
Female (*N* = 190)	1.41 (0.50)	92.6 [88.9, 96.4]	7.4 [3.6, 11.1]	0.0
Male (*N* = 195)	1.37 (0.56)	91.8 [87.9, 95.7]	8.2 [4.3, 12.1]	0.0
Asian or Asian British (*N* = 65)	1.37 (0.60)	87.7 [79.5, 95.9]	12.3 [4.1, 20.5]	0.0
Black African, Caribbean, or Black British (*N* = 21)	1.33 (0.47)	95.2 [85.3, 100.1]	4.8 [−5.2, 14.7]	0.0
Mixed or multiple ethnicities (*N* = 12)	1.39 (0.55)	91.7 [73.3, 110.0]	8.3 [−10.0, 26.7]	0.0
White (English, Scottish, Welsh, Irish, or any other White background) (*N* = 291)	1.41 (0.54)	93.1 [90.2, 96.1]	6.5 [3.7, 9.4]	0.3 [0.3, 1.0]

Mental health was measured with the PHQ‐4, a short version of the Patient Health Questionnaire [68]. The PHQ‐4 is a 4‐item validated screener for general anxiety and depression. Participants were asked to report if they had experienced any symptoms of anxiety or depression over the prior 2 weeks (e.g., “feeling nervous, anxious or on edge”). Although the PHQ‐4 is not strictly speaking a measure of mental health, we adopted it as an indicator of mental health in this study because anxiety and depression are the most commonly diagnosed mental health problems in the UK. Higher PHQ‐4 scores indicate stronger symptoms of anxiety and depression (i.e., poorer mental health).

Insomnia severity was measured with the Insomnia Severity Index (ISI) [69]. The ISI is a 7‐item scale that measures the nature, severity, and impact of insomnia over a period of 2 weeks. The scale items map onto seven components: severity of sleep onset, sleep maintenance, early morning awakening, sleep dissatisfaction, interference of sleep difficulties with daytime functioning, noticeability of sleep problems by others, and distress caused by sleep difficulties (e.g., “To what extent do you consider your sleep problem to interfere with your daily functioning [daytime fatigue, ability to function at work/daily chores, concentration, mood, etc.]?”). Responses to the items were recorded with a 5‐point Likert scale. Higher ISI scores represent poorer quality of sleep.

Financial anxiety was measured with the Financial Anxiety Scale (FAS) [70]. The scale comprises 7 items. Example items include: “I feel anxious about my financial situation,” “I am irritable because of my financial situation,” and “I have difficulty sleeping because of my financial situation.” Responses were recorded on a 5‐point scale ranging from “never” to “always.”

Loneliness was measured with the 3‐item UCLA short scale for measuring loneliness [71]. An example of constituent items is: “How often do you feel isolated from others?” Responses were recorded on a 3‐point scale ranging from “hardly ever” to “often.”

Health Anxiety was measured with the Short Health Anxiety Inventory (SHAI) [72]. The scale comprises 18 items measuring two dimensions of health anxiety and hypochondriasis: illness likelihood and illness severity. Example items include: “I am aware of aches and pains in my body all the time,” and “resisting thoughts of illness is never a problem (reversed).”

Worry about the COVID‐19 pandemic was measured with the 4‐item coronavirus risk perception scale [73]. Example items include: “Are you worried about your family getting infected with COVID‐19?”. Responses were recorded on a 5‐point scale ranging from “strongly worried” to “not worried at all.”

The Russian invasion of Ukraine began in February 2022. During data collection for this study, we decided to account for any potential psychological impacts of the crisis in our analyses. Worry about the Russia‐Ukraine War was, therefore, measured among the general population sample with a single question: “Are you worried about the Russian invasion of Ukraine?” Answers were provided on a 5‐point rating scale (1 = not worried at all, 5 = very worried). This question was only asked in the survey administered to the general UK resident population sample.

Demographic covariates—participants’ age and gender—were also measured in the study. The choice of covariates is justified by evidence of greater prevalence of depression, anxiety disorders, and insomnia among females and younger adults [74, 75]. Descriptive statistics and reliability estimates for the measures are presented in Table 2.

**TABLE 2:** Descriptive and reliability statistics for psychometric measures used in the study.

	Student population (*N* = 461)			General population (*N* = 400)		
	M (SD)	α	Range	M (SD)	α	Range
Climate anxiety	1.40 (0.54)	0.93	1–5	1.40 (0.54)	0.93	1–5
Financial anxiety	1.99 (0.96)	0.94	1–5	2.09 (0.95)	0.93	1–5
Health anxiety	2.03 (0.44)	0.88	1–4	1.98 (0.50)	0.91	1–4
Loneliness	1.94 (0.63)	0.81	1–3	1.96 (0.63)	0.83	1–3
COVID‐19 worry	2.99 (0.90)	0.82	1–5	2.72 (1.09)	0.89	1–5
Insomnia severity	2.46 (0.75)	0.83	1–5	1.38 (0.75)	0.83	0–4
Mental health	2.26 (0.88)	0.89	1–4	2.27 (0.84)	0.88	1–4
Worry about Russia‐Ukraine war				4.09 (.97)	—	1–5

Abbreviations: M, mean; SD, standard deviation.

### 2.3 Preregistration Notes and Deviations

There were a few deviations from the preregistered plan for this study:

1. The preregistration does not cover data from the general UK resident population sample, as this component of the study was only added after the preregistration had been submitted.
2. The order of hypotheses and research questions has been changed in this article to benefit narrative flow. H1 and H2 correspond to H2a and H1b, respectively, in the preregistration. RQ1 and RQ2 correspond with RQ3 and RQ2, respectively, in the preregistration.
3. The preregistration included hypotheses relating climate anxiety to sleep quality (measured with the Pittsburgh Sleep Quality Inventory [PSQI]) and insomnia severity (measured with the Insomnia Severity Inventory [ISI]). PSQI and ISI scores were strongly correlated (student sample: *r* = 0.70, *p* <0.001; general UK population sample: *r* = 0.68, *p* <0.001). Multiple regression of PSQI scores on CCAS and other psychological stressors are provided as Supplementary Data (see File S1). For the sake of concision, we only report results relating to insomnia severity in this article.
4. The preregistration includes hypotheses regarding “negative emotional responses to climate change” as a predictor of mental health and insomnia severity (H1c, H1d, and H2b). This variable was measured with a scale considered to capture emotion‐based climate anxiety [76, 77], as opposed to the CCAS, which captures symptoms of cognitive‐emotional and functional impairment. Emotion‐based climate anxiety is moderately correlated with CCAS [48, 77]. Considering that the CCAS is the more widely used index of climate anxiety, we decided to focus only on the CCAS in this article to enable direct comparability with a broader range of studies. Nonetheless, the main findings were also replicated using the emotion‐based climate anxiety measure (see File S2).
5. The preregistration states that our hypotheses (H1 and H2) would be tested using multiple regression. On recommendation from an anonymous reviewer, we used structural equation modeling (SEM) with latent variables to test the hypotheses and to explore the research questions. This approach provides more robust estimates by accounting for measurement error. The results obtained using multiple regression are provided as Supplementary Data (File S1).
6. The preregistration did not explicitly state how we planned to compare the regression coefficients for climate anxiety with those of health anxiety, financial anxiety, loneliness, and COVID‐19 risk perception as predictors of mental health and insomnia. We used dominance analysis to compare the predictors because it is an appropriate method for determining the relative importance of individual predictors in a regression model while accounting for the correlations among predictors [78].

### 2.4 Analysis

Data preparation and calculation of descriptive statistics and zero‐order intercorrelations among variables were conducted using SPSS version 27. SEM was conducted with R software [79] using the *lavaan* package [80]. Latent variable regression models were used to concurrently assess climate anxiety and the other psychological stressors as predictors of mental health and insomnia severity while controlling for age and gender. Details of the SEM regression model specifications, including illustrative path diagrams, are provided in File S3. Without modification, the SEM regression models showed acceptable fit to the data based on conventional thresholds for determining good fit (RMSEA < 0.06, SRMR < 0.08, CFI > 0.90) [81].

Dominance analysis [82] was conducted on the latent variable regression models using the *misty* [83] package in R. This technique determines the relative dominance of each predictor based on its average contribution to the model's explanatory power (*R* ^2^) across all possible combinations of predictors [84]. When we state that one predictor dominates another, this means that it contributes more to the model's predictive power consistently across most or all subset models. Dominance analysis is increasingly used in psychological research [85], and allows for model‐independent conclusions to be drawn about the statistical importance of each predictor, which is otherwise not possible using multiple regression [86]. The data and analysis code underlying this article are available on OSF (https://osf.io/eycbm/).

## 3 Results

### 3.1 Student Population

Climate anxiety showed a small but significant positive zero‐order correlation with poor mental health (Table 3). It was also positively correlated with insomnia severity. Significant partial correlations of climate anxiety with mental health (*r* = 0.17, *p* < 0.001) and insomnia severity (*r* = 0.53, *p* <0.001) were also observed when age and gender differences were controlled. Therefore, at this level of analysis, our hypotheses (H1) and (H2) were supported among the student sample.

**TABLE 3:** Zero‐order correlations matrix (student sample).

	2	3	4	5	6	7
1. Climate anxiety	0.37***	0.29***	0.13**	0.26***	0.28***	0.19***
2. Financial anxiety		0.40***	0.22***	0.18***	0.47***	0.42***
3. Health anxiety			0.27***	0.28***	0.35***	0.45***
4. Loneliness				0.09	0.31***	0.48***
5. COVID‐19 worry					0.15**	0.22***
6. Insomnia severity						0.54***
7. Mental health						—

*Note*: Cell entries are Pearson product‐moment correlation coefficients.

**p < 0.01; ***p < 0.001.

However, climate anxiety was assessed alongside other psychological stressors: financial anxiety, health anxiety, loneliness, and worry about COVID‐19 using an SEM. The relationship between climate anxiety and mental health was nonsignificant (Table 4). Meanwhile, the relationship between climate anxiety and insomnia severity remained significant even when other stressors were included in the model (Table 5).

**TABLE 4:** Structural equation model results of mental health regressed on climate anxiety, with dominance analysis ranking (student sample).

	B	SE_B_	β	*z*	*p*	95% CI of *B*		*R* ^2^	Ranking
						Lower	Upper		
Climate anxiety	−0.09	0.07	−0.06	−1.24	0.216	−0.23	0.05	0.008	5
Loneliness	0.79	0.10	0.41	7.60	<0.001	0.58	0.99	0.214	1
Financial anxiety	0.19	0.05	0.20	4.11	<0.001	0.10	0.28	0.080	3
Health anxiety	0.63	0.13	0.27	5.01	<0.001	0.38	0.88	0.129	2
COVID‐19 worry	−0.10	0.05	−0.09	−1.82	0.068	−0.20	0.01	0.026	4
Age	−0.03	0.03	−0.04	−1.01	0.311	−0.08	0.03	0.001	7
Gender (Male)	−0.13	0.11	−0.05	−1.18	0.239	−0.35	0.09	0.004	6
Model fit: χ^2^(1054) = 2521.78, *p* <0.001; CFI = 0.862, RMSEA = 0.056, SRMR = 0.056, *R* ^2^ = 0.463.									

*Note*: N = 446, Gender was coded as female = 0, male = 1. Predictor ranking is based on average contribution to the model's *R* ^2^ across all possible combinations of predictors.

**TABLE 5:** Insomnia severity regressed on climate anxiety, with dominance analysis ranking (student sample).

	B	SE_B_	β	*z*	*p*	95% CI of *B*		*R* ^2^	Ranking
						Lower	Upper		
Climate anxiety	0.15	0.06	0.12	2.33	0.020	0.02	0.27	0.042	4
Loneliness	0.31	0.08	0.20	3.82	<0.001	0.15	0.47	0.069	3
Financial anxiety	0.24	0.04	0.32	5.52	<0.001	0.15	0.32	0.135	1
Health anxiety	0.32	0.11	0.17	2.99	0.003	0.11	0.52	0.070	2
COVID‐19 worry	0.02	0.05	0.02	0.32	0.747	−0.08	0.11	0.006	5
Age	0.00	0.02	0.00	0.01	0.991	−0.05	0.05	0.002	6
Gender (Male)	0.02	0.10	0.01	0.17	0.866	−0.18	0.21	0.000	7
Model fit: χ^2^(1198) = 2731.26, *p* <0.001; CFI = 0.857, RMSEA = 0.054, SRMR = 0.057, *R* ^2^ = 0.324.									

*Note*: N = 446, Gender was coded as female = 0, male = 1. Predictor ranking is based on average contribution to the model's *R* ^2^ across all possible combinations of predictors.

To address our research question (RQ1), we compared the relative importance of the predictor variables to the mental health outcome using dominance analysis. This revealed that loneliness was the most dominant predictor of mental health among the student sample, followed by health anxiety, financial anxiety, COVID‐19 worry, and lastly, climate anxiety (see Table 4 for predictor rankings). Climate anxiety was not a significant predictor of mental health when the other stressors were considered, which contradicts our hypothesis (H1). It was also the least dominant of the psychological stressors assessed. When the demographic factors (age, gender) and other psychological stressors are controlled, the average contribution of climate anxiety to predicting poorer mental health among the student sample equated to less than 1% explained variance.

We also compared the relative importance of the predictor variables to insomnia severity (RQ2: Table 5). The dominance analysis showed that financial anxiety was the most dominant predictor of insomnia severity, followed by health anxiety and loneliness. Climate anxiety ranked fourth out of five psychological stressors in relative importance as a predictor of insomnia severity. Overall, the dominance analysis showed that after accounting for demographic factors (age, gender) and the other psychological stressors, the contribution of climate anxiety to predicting insomnia severity among the student sample was equivalent to approximately 4% explained variance.

### 3.2 General Population

Similar to the student sample, climate anxiety had significant positive zero‐order correlations with poor mental health and insomnia severity among the general population sample (Table 6). The correlation of climate anxiety with mental health (*r* = 0.24, *p* <0.001) and insomnia severity (*r* = 0.19, *p* <0.001) remained significant even after controlling for age and gender. However, when other psychological stressors were controlled for using SEM, the association of climate anxiety with mental health (Table 7) and insomnia severity (Table 8) became nonsignificant.

**TABLE 6:** Zero‐order correlations between mental health, sleep quality, climate anxiety, and other psychological stressors (general population sample).

	2	3	4	5	6	7	8
Climate anxiety	0.36***	0.36***	0.20***	0.30***	0.16**	0.21**	0.27***
Financial anxiety		0.44***	0.33***	0.21***	0.10*	0.43***	0.54***
Health anxiety			0.41***	0.43***	0.19***	0.38***	0.52***
Loneliness				0.15**	0.00	0.38***	0.57***
COVID‐19 worry					0.44***	0.28***	0.27***
Worry about Russia‐Ukraine war						0.06	0.14**
Insomnia severity							0.55***
Mental health							—

*Note*: N = 400. Cell entries are Pearson correlation coefficients.

*p < 0.05, **p < 0.01; ***p < 0.001.

**TABLE 7:** Mental health regressed on climate anxiety, with dominance analysis ranking (general population sample).

	B	SE_B_	β	*z*	*p*	95% CI of *B*		*R* ^2^	Ranking
						Lower	Upper		
Climate anxiety	−0.11	0.06	−0.08	−1.83	0.068	−0.23	0.01	0.014	5
Loneliness	0.72	0.10	0.41	7.45	<0.001	0.53	0.91	0.231	1
Financial anxiety	0.30	0.05	0.31	5.84	<0.001	0.20	0.40	0.153	2
Health anxiety	0.46	0.11	0.24	4.11	<0.001	0.24	0.68	0.135	3
COVID‐19 worry	0.03	0.06	0.03	0.53	0.593	−0.08	0.14	0.022	4
Ukraine war worry	0.05	0.04	0.06	1.37	0.170	−0.02	0.13	0.008	7
Age	−0.03	0.02	−0.07	−1.79	0.074	−0.06	0.00	0.005	8
Gender (Male)	−0.10	0.07	−0.06	−1.41	0.160	−0.23	0.04	0.013	6
Model fit: χ^2^(1094) = 2584.12, *p*<0.001; CFI = 0.861, RMSEA = 0.059, SRMR = 0.059, *R* ^2^ = 0.580.									

*Note*: N = 385, Gender was coded as female = 0, male = 1. Predictor ranking is based on average contribution to the model's *R* ^2^ across all possible combinations of predictors.

**TABLE 8:** Insomnia severity regressed on climate anxiety, with dominance analysis ranking (general population sample).

	B	SE_B_	β	*z*	*p*	95% CI of *B*		*R* ^2^	Ranking
						Lower	Upper		
Climate anxiety	−0.06	0.05	−0.07	−1.20	0.231	−0.16	0.04	0.009	6
Loneliness	0.31	0.08	0.25	4.08	<0.001	0.16	0.45	0.097	2
Financial anxiety	0.20	0.04	0.31	4.71	<0.001	0.12	0.29	0.112	1
Health anxiety	0.12	0.09	0.09	1.34	0.180	−0.06	0.29	0.056	3
COVID‐19 worry	0.15	0.05	0.20	3.10	0.002	0.05	0.24	0.043	4
Ukraine war worry	−0.02	0.03	−0.03	−0.57	0.571	−0.08	0.04	0.003	7
Age	−0.03	0.01	−0.12	−2.43	0.015	−0.06	−0.01	0.013	5
Gender (Male)	0.05	0.06	0.04	0.87	0.383	−0.06	0.16	0.002	8
Model Fit: χ^2^(1241) = 2714.60, *p* < 0.001; CFI = 0.859, RMSEA = 0.056, SRMR = 0.062, *R* ^2^ = 0.335.									

*Note*: N = 385. Gender was coded as female = 0, male = 1. Predictor ranking is based on average contribution to the model's *R* ^2^ across all possible combinations of predictors.

The dominance analysis again showed that loneliness was the most dominant predictor of mental health among the general population sample (Table 7). This was followed by financial anxiety and health anxiety. Climate anxiety ranked fourth out of six psychological stressors assessed in terms of contribution to predicting mental health among the general population sample. After controlling for demographics and the other psychological stressors, the average contribution of climate anxiety to predicting poor mental health among the general population sample of UK young adults equated to approximately 1% of explained variance.

Replicating findings from the student sample, the dominance analysis also showed that financial anxiety was the most dominant predictor of insomnia severity among the general population sample, followed by loneliness and health anxiety (Table 8). Climate anxiety was the fifth most important predictor of insomnia severity among the general population sample, out of six psychological stressors assessed. The average contribution of climate anxiety to predicting insomnia severity among the general population sample of UK young adults, after accounting for demographics and the other psychological stressors, equated to approximately 1% of explained variance in insomnia severity.

## 4 Discussion

Our analysis is the first to show that, after accounting for other common psychological stressors, specifically loneliness, financial, and health anxiety, climate anxiety shows little predictive power in explaining mental health and sleep disturbance among young people. While there is little doubt that climate anxiety is a growing stressor in the lives of young people [4, 16], we argue that a more realistic assessment of the significance of emotional responses to climate change can be obtained by contrasting them with other common psychological stressors. Below, we offer some potential explanations for the comparative importance of climate anxiety as a predictor of mental health and wellbeing relative to other stressors.

### 4.1 Construal Level Theory

In line with Construal Level Theory [87], the results of this study may reflect the fact that climate change tends to be perceived as psychologically distant and abstract relative to more proximal, concrete threats. For example, loneliness, financial, and health anxieties may be perceived as more immediate threats to one's daily wellbeing and quality of life compared with climate change, which is often perceived as temporally distant [88]. Indeed, when asked in 2024 to state the most important issues facing the UK, 57% of British adults mentioned climate change compared with 85% who mentioned the state of the National Health Service and 84% who mentioned the cost‐of‐living crisis [89]. In this study, fewer than 1 in 10 respondents exhibited levels of climate anxiety that could be described as moderate or severe.

The social proximity of stressors may further explain why climate anxiety did not emerge as a dominant predictor of mental health and sleep, relative to other stressors. Research shows that the closeness or relative personal impact of a risk is related to greater concern and increased threat perception [90]. Stressors like loneliness, financial, and health anxieties, which pose a personal‐level threat, have a greater role in mental health than climate change, which may be perceived as a more diffuse, societal‐level threat.

### 4.2 Finite Pool of Worry

Some scholars argue that humans have finite emotional resources, whereby worry about one threat may diminish worry for another [91]. For example, there is some evidence that worry about climate change, online discussions of climate change, and attention to climate change‐related media decreased during the COVID‐19 pandemic [92, 93, 94]. Others show that climate change worry decreases during armed conflicts [95]. In the restrictive conditions induced by COVID‐19, young people experienced heightened loneliness, health, and financial anxiety [96], which may have shifted their attention from climate‐related anxieties. This is in line with previous observations that competing worries about pressing daily concerns may dilute climate anxiety, especially among those experiencing strong challenges and adversity in other areas of life [97]. It is important to note, however, that worry is a different concept from anxiety [98], and evidence for the finite pool of the worry hypothesis has been mixed [93, 99].

### 4.3 Contextualizing Climate Anxiety

Our findings suggest that climate anxiety is a less important predictor of mental health and sleep among young adults than common psychological stressors like loneliness, financial anxiety, and health anxiety. This provides a more realistic perspective than may be obtained when climate anxiety is analyzed in isolation. However, it is important to note that these stressors may be overlapping and interconnected. For example, loneliness and perceived social disconnectedness have previously been linked with heightened climate anxiety [100, 101]. Concern about health impacts is also an important frame for heightening climate worry and motivating action [102]. This means that climate anxiety could contribute to loneliness and social disconnection when young people do not feel listened to or feel that others have a shared understanding of the climate threat. Health and financial anxieties may also feed into climate anxiety by amplifying worries about the impacts of climate change on future health and financial security. Current measures of climate anxiety do not capture these potential interrelations between stressors, nor do they consider how climate anxiety could manifest as worries about one's relatedness with others (e.g., shared values and understanding), or worry about future health and finances. In the current study, we observed that climate anxiety only showed small to moderate correlations with the other stressors. Systematic efforts to unveil the interrelationships among concurrent psychological stressors and better contextualize climate anxiety in the social and developmental dynamics of the young adult life stage is a critically important direction for future research.

### 4.4 Limitations of the Study

Given the low prevalence of individuals experiencing moderate or severe climate anxiety among our participant samples (<10%), our findings may not generalize to groups of young people who are experiencing severe climate anxiety. For such groups, it is possible that worries about the climate crisis are a dominant predictor of mental health, meaning that targeting climate anxiety is a justifiable priority for mental health and resilience promotion efforts in those specific instances [58]. Additionally, due to the cross‐sectional design of the study, our data cannot establish the direction of causal relationships between mental health, insomnia severity, climate anxiety, and the other psychological stressors. Instead, the value of this study lies partly in identifying patterns for future causal/longitudinal research.

## 5 Summary and Conclusions

This study is the first to contextualize the predictive importance of climate anxiety in young people's mental health and wellbeing, relative to other psychological stressors. Notably, we find that tangible, immediate, and personal concerns such as loneliness, financial anxiety, and health anxiety are more dominant predictors of mental health and insomnia severity than climate anxiety. The replication of the results across two distinct samples (university students and the age‐matched general UK resident population), and for both mental health and insomnia severity, underscores the reliability of our findings. For the first time, novel analytical approaches quantified the relative importance of relevant psychological stressors as concurrent predictors of mental health. Despite this novelty, the stressors considered are not exhaustive, and future work should explore other prominent stressors relative to climate anxiety, including educational‐, family‐, and appearance‐related anxieties. Importantly, our research highlights the need for greater attention to the interconnected nature of social, economic, and ecological stressors in attempts to map pathways linking climate change with mental health.

## Author Contributions

C.A.O., S.L., M.S.‐A.P., and R.D. conceptualized the study. C.A.O., S.L., and M.S.‐A.P. gathered the data. C.A.O. and L.P. conducted the data analysis, summarized findings, and prepared the tables. C.A.O. prepared the first draft of the manuscript with input from L.P. All authors reviewed the manuscript and contributed to subsequent drafts of the paper during the revision process. C.A.O. had full access to the data and had final responsibility for the decision to submit for publication.

## Conflicts of Interest

The authors declare no competing interests.

## Supporting information




**Supporting Material**: nyas70057‐sup‐0001‐SuppFileS1.docx

**Supporting Material**: nyas70057‐sup‐0002‐SuppFileS2.docx

**Supporting Material**: nyas70057‐sup‐0003‐SuppFileS3.docx

## Data Availability

The data that support the findings of this study are openly available in OSF at https://osf.io/eycbm/, reference number DOI 10.17605/OSF.IO/EYCBM.

## Note

1 Gen Z refers to the cohort of people born between 1997 and 2012.
