# Supplementary material for: Climate Anxiety in Perspective: A Look at Dominant Stressors in Youth Mental Health and Sleep
Source: Ann N Y Acad Sci. 2025 Sep 15;1552(1):249–62. doi: 10.1111/nyas.70057 (PMC12576874; doi:10.1111/nyas.70057)
Supplement: Supplementary file 1 — Supporting Material: nyas70057‐sup‐0001‐SuppFileS1.docx [file NYAS-1552-249-s001.docx]

**Supporting File S1 – OLS regression results**

**Table S1**. Mental health regressed on climate anxiety, with dominance analysis ranking (student sample).

|  | *B* | *SE_B_* | *β* | *t* | *p* | *sr_2_* | 95% BCa CI | | GDW | Ranking |
| --- | --- | --- | --- | --- | --- | --- | --- | --- | --- | --- |
|  |  |  |  |  |  |  | Lower | Upper |  |  |
| Climate anxiety | 0.07 | 0.07 | 0.04 | 1.03 | 0.302 | 0.001 | -0.07 | 0.19 | 0.009 | 5 |
| Health anxiety | -0.48 | 0.09 | -0.24 | -5.65 | < .001 | 0.044 | -0.67 | -0.31 | 0.105 | 2 |
| Financial anxiety | -0.22 | 0.04 | -0.24 | -5.74 | < .001 | 0.045 | -0.30 | -0.15 | 0.094 | 3 |
| Loneliness | -0.51 | 0.05 | -0.36 | -9.37 | < .001 | 0.120 | -0.61 | -0.41 | 0.169 | 1 |
| COVID-19 worry | -0.08 | 0.04 | -0.09 | -2.26 | 0.024 | 0.007 | -0.16 | -0.01 | 0.020 | 4 |
| Age | 0.03 | 0.03 | 0.04 | 1.15 | 0.250 | 0.002 | -0.03 | 0.09 | 0.001 | 7 |
| Gender (Male) | 0.11 | 0.11 | 0.04 | 1.01 | 0.311 | 0.001 | -0.08 | 0.32 | 0.004 | 6 |
| Model Fit: *F*(7, 446) = 42.21, *p* < .001, *R^2^ =* 0.402, *R^2^_adj_* = 0.393 | | | | | | | | | | |

*Note.* Gender was coded as female = 0, male = 1. 95% bootstrapped bias-corrected confidence intervals (95% BCa CI) with 1,000 resamples are reported. *sr_2_ =* squared semi-partial correlation._._ Ranking of predictors is based on General Dominance Weights (GDW).

**Table S2.** Insomnia severity regressed on climate anxiety, with dominance analysis ranking (student sample).

|  | *B* | *SE_B_* | *β* | *t* | *p* | *sr_2_* | 95% BCa CI | | GDW | Ranking |
| --- | --- | --- | --- | --- | --- | --- | --- | --- | --- | --- |
|  |  |  |  |  |  |  | Lower | Upper |  |  |
| Climate anxiety | 0.12 | 0.06 | 0.09 | 1.91 | 0.057 | 0.006 | 0.00 | 0.26 | 0.031 | 4 |
| Health anxiety | 0.23 | 0.08 | 0.14 | 2.91 | 0.004 | 0.014 | 0.08 | 0.39 | 0.053 | 3 |
| Financial anxiety | 0.27 | 0.04 | 0.34 | 7.27 | < .001 | 0.086 | 0.19 | 0.34 | 0.138 | 1 |
| Loneliness | 0.21 | 0.05 | 0.18 | 4.19 | < .001 | 0.029 | 0.11 | 0.32 | 0.053 | 2 |
| COVID-19 worry | 0.00 | 0.03 | 0.01 | 0.12 | 0.907 | 0.000 | -0.06 | 0.08 | 0.005 | 5 |
| Age | 0.01 | 0.02 | 0.02 | 0.35 | 0.724 | 0.000 | -0.04 | 0.06 | 0.003 | 6 |
| Gender (Male) | 0.01 | 0.10 | 0.01 | 0.15 | 0.882 | 0.000 | -0.18 | 0.22 | 0.001 | 7 |
| Model Fit: *F*_(7, 446)_ = 24.79, *p* < .001, *R^2^ =* 0.283 *R^2^_adj_* = 0.272 | | | | | | | | | | |

*Note.* Gender was coded as female = 0, male = 1. 95% bootstrapped bias-corrected confidence intervals (95% BCa CI) with 1,000 resamples are reported. *sr_2_ =* squared semi-partial correlation. Ranked predictors are based on General Dominance Weights (GDW).

**Table S3.** *Mental health regressed on climate anxiety, with dominance analysis ranking (general population sample).*

|  | *B* | *SE_B_* | *β* | *t* | *p* | *sr_2_* | 95% BCa CI | | GDW | Ranking |
| --- | --- | --- | --- | --- | --- | --- | --- | --- | --- | --- |
|  |  |  |  |  |  |  | Lower | Upper |  |  |
| Climate anxiety | 0.02 | 0.06 | 0.01 | 0.36 | .721 | 0.000 | -0.10 | 0.15 | 0.016 | 4 |
| Health anxiety | -0.32 | 0.08 | -0.19 | -4.09 | < .001 | 0.022 | -0.48 | -0.17 | 0.111 | 3 |
| Financial anxiety | -0.28 | 0.04 | -0.31 | -7.35 | < .001 | 0.071 | -0.36 | -0.20 | 0.145 | 2 |
| Loneliness | -0.52 | 0.06 | -0.39 | -9.46 | < .001 | 0.117 | -0.63 | -0.41 | 0.200 | 1 |
| COVID-19 worry | -0.02 | 0.04 | -0.03 | -0.56 | .577 | 0.000 | -0.09 | 0.05 | 0.016 | 5 |
| Ukraine war worry | -0.04 | 0.04 | -0.04 | -1.05 | .294 | 0.001 | -0.11 | 0.04 | 0.005 | 7 |
| Age | 0.03 | 0.02 | 0.07 | 1.85 | .066 | 0.004 | 0.00 | 0.06 | 0.005 | 8 |
| Gender (Male) | 0.09 | 0.06 | 0.06 | 1.45 | .147 | 0.003 | -0.03 | 0.22 | 0.010 | 6 |
| Model Fit: *F*_(8, 384)_ = 48.52, *p* < .001, *R^2^ =* 0.508, *R^2^_adj_* = 0.497 | | | | | | | | | | |

*Note.* Gender was coded as female = 0, male = 1. 95% bootstrapped bias-corrected confidence intervals (95% BCa CI) with 1,000 resamples are reported. *sr_2_ =* squared semi-partial correlation. Ranking of predictors is based on General Dominance Weights (GDW).

**Table S4.** *Insomnia severity regressed on climate anxiety, with dominance analysis ranking (general population sample).*

|  | *B* | *SE_B_* | *β* | *t* | *p* | *sr_2_* | 95% BCa CI | | GDW | Ranking |
| --- | --- | --- | --- | --- | --- | --- | --- | --- | --- | --- |
|  |  |  |  |  |  |  | Lower | Upper |  |  |
| Climate anxiety | -0.03 | 0.07 | -0.02 | -0.44 | .661 | 0.000 | -0.17 | 0.12 | 0.010 | 6 |
| Health anxiety | 0.12 | 0.09 | 0.08 | 1.45 | .148 | 0.004 | -0.04 | 0.31 | 0.050 | 3 |
| Financial anxiety | 0.25 | 0.04 | 0.31 | 6.12 | < .001 | 0.070 | 0.16 | 0.33 | 0.111 | 1 |
| Loneliness | 0.28 | 0.06 | 0.23 | 4.68 | < .001 | 0.041 | 0.16 | 0.40 | 0.080 | 2 |
| COVID-19 worry | 0.11 | 0.04 | 0.16 | 3.03 | .003 | 0.017 | 0.04 | 0.19 | 0.032 | 4 |
| Ukraine war worry | -0.01 | 0.04 | -0.01 | -0.28 | .779 | 0.000 | -0.08 | 0.06 | 0.002 | 7 |
| Age | -0.04 | 0.02 | -0.11 | -2.59 | .010 | 0.013 | -0.07 | -0.01 | 0.012 | 5 |
| Gender (Male) | 0.03 | 0.07 | 0.02 | 0.37 | .713 | 0.000 | -0.11 | 0.16 | 0.002 | 8 |
| Model Fit: *F*_(8, 384)_ = 20.07, *p* < .001, *R^2^ =* 0.299, *R^2^_adj_* = 0.284 | | | | | | | | | | |

*Note.* Gender was coded as female = 0, male = 1. 95% bootstrapped bias-corrected confidence intervals (95% BCa CI) with 1,000 resamples are reported. *sr_2_ =* squared semi-partial correlation. Ranked predictors are based on General Dominance Weights (GDW).

**Table S5.** *Sleep quality regressed on climate anxiety, with dominance analysis ranking (student sample).*

|  | *B* | *SE_B_* | *β* | *t* | *p* | *sr_2_* | 95% CI | | GDW | Ranking |
| --- | --- | --- | --- | --- | --- | --- | --- | --- | --- | --- |
|  |  |  |  |  |  |  | Lower | Upper |  |  |
| Climate anxiety | 0.65 | 0.26 | 0.11 | 2.47 | .014 | 0.014 | 0.13 | 1.17 | 0.032 | 4 |
| Health anxiety | 0.62 | 0.34 | 0.09 | 1.80 | .073 | 0.007 | -0.06 | 1.28 | 0.034 | 3 |
| Financial anxiety | 0.84 | 0.16 | 0.26 | 5.40 | <.001 | 0.062 | 0.54 | 1.15 | 0.087 | 1 |
| Loneliness | 1.28 | 0.22 | 0.26 | 5.87 | <.001 | 0.073 | 0.85 | 1.71 | 0.085 | 2 |
| COVID-19 worry | 0.03 | 0.15 | 0.01 | 0.16 | .870 | 0.000 | -0.28 | 0.33 | 0.005 | 6 |
| Age | -0.13 | 0.11 | -0.05 | -1.22 | .223 | 0.003 | -0.34 | 0.08 | 0.002 | 7 |
| Gender (Male) | -0.74 | 0.42 | -0.07 | -1.75 | .081 | 0.007 | -1.56 | 0.09 | 0.005 | 5 |
| Model Fit: *F*_(7, 439)_ = 20.82, *p* < .001, *R^2^ =* 0.249, *R^2^_adj_* = 0.237 | | | | | | | | | | |

*Note.* Gender was coded as female = 0, male = 1. *sr_2_ =* squared semi-partial correlation. Ranked predictors are based on General Dominance Weights (GDW).

**Table S6.** *Sleep quality regressed on climate anxiety, with dominance analysis ranking (general population sample).*

|  | *B* | *SE_B_* | *β* | *t* | *p* | *sr_2_* | 95% CI | | GDW | Ranking |
| --- | --- | --- | --- | --- | --- | --- | --- | --- | --- | --- |
|  |  |  |  |  |  |  | Lower | Upper |  |  |
| Climate anxiety | -0.55 | 0.36 | -0.08 | -1.52 | .129 | 0.006 | -1.25 | 0.16 | 0.006 | 5 |
| Health anxiety | 0.66 | 0.45 | 0.09 | 1.47 | .142 | 0.006 | -0.22 | 1.54 | 0.039 | 3 |
| Financial anxiety | 1.13 | 0.21 | 0.29 | 5.47 | <.001 | 0.074 | 0.73 | 1.54 | 0.095 | 1 |
| Loneliness | 1.33 | 0.30 | 0.22 | 4.43 | <.001 | 0.050 | 0.74 | 1.92 | 0.072 | 2 |
| COVID-19 worry | 0.27 | 0.17 | 0.08 | 1.56 | .121 | 0.006 | -0.07 | 0.61 | 0.014 | 4 |
| Ukraine war worry | -0.03 | 0.18 | -0.01 | -0.15 | .882 | 0.000 | -0.38 | 0.33 | 0.000 | 8 |
| Age | 0.05 | 0.08 | 0.03 | 0.64 | .522 | 0.001 | -0.11 | 0.21 | 0.001 | 7 |
| Gender (Male) | -0.39 | 0.35 | -0.05 | -1.12 | .265 | 0.003 | -1.07 | 0.30 | 0.005 | 6 |
| Model Fit: *F*_(8, 376)_ = 14.11, *p* < .001, *R^2^ =* 0.231, *R^2^_adj_* = 0.215 | | | | | | | | | | |

*Note.* Gender was coded as female = 0, male = 1. *sr_2_ =* squared semi-partial correlation. Ranked predictors are based on General Dominance Weights (GDW).
