# Supplementary material for: Climate Anxiety in Perspective: A Look at Dominant Stressors in Youth Mental Health and Sleep
Source: Ann N Y Acad Sci. 2025 Sep 15;1552(1):249–62. doi: 10.1111/nyas.70057 (PMC12576874; doi:10.1111/nyas.70057)
Supplement: Supplementary file 2 — Supporting Material: nyas70057‐sup‐0002‐SuppFileS2.docx [file NYAS-1552-249-s003.docx]

**Supporting File S2 – SEM regression of mental health and insomnia severity on emotion-based climate anxiety and other psychological stressors**

Emotion-based climate anxiety was measured using a scale adopted from Ogunbode et al.

[add details about scale items and reliability across samples]

**SF2 - Table S1**. SEM results of mental health regressed on emotion-based climate anxiety, with dominance analysis ranking (student sample).

|  | B | SE_B_ | β | z | p | 95% CI of *B* | | *R*^2^ | Ranking |
| --- | --- | --- | --- | --- | --- | --- | --- | --- | --- |
|  |  |  |  |  |  | Lower | Upper |  |  |
| Climate anxiety | 0.01 | 0.06 | 0.01 | 0.19 | .848 | -0.10 | 0.13 | .001 | 7 |
| Loneliness | 0.79 | 0.10 | 0.41 | 7.61 | <.001 | 0.58 | 0.99 | .215 | 1 |
| Financial anxiety | 0.17 | 0.04 | 0.19 | 3.96 | <.001 | 0.09 | 0.26 | .081 | 3 |
| Health anxiety | 0.61 | 0.12 | 0.27 | 4.92 | <.001 | 0.37 | 0.86 | .131 | 2 |
| COVID-19 worry | -0.09 | 0.05 | -0.08 | -1.66 | .097 | -0.19 | 0.02 | .027 | 4 |
| Age | -0.03 | 0.03 | -0.04 | -1.08 | .280 | -0.09 | 0.03 | .001 | 6 |
| Gender (Male) | -0.12 | 0.11 | -0.04 | -1.06 | .289 | -0.34 | 0.10 | .005 | 5 |
| Model fit: χ²(793) =2119.01, *p* <.001; CFI = 0.845, RMSEA = 0.061, SRMR = 0.056, *R*^2^ = 0.462. | | | | | | | | | |

*Note.* *N* = 447, Gender was coded as female = 0, male = 1. Predictor ranking is based on average contribution to the model’s *R*^2^ across all possible combinations of predictors.

**SF2 - Table S2**. SEM results of insomnia severity regressed on emotion-based climate anxiety, with dominance analysis ranking (student sample).

|  | B | SE_B_ | β | z | p | 95% CI of *B* | | *R*^2^ | Ranking |
| --- | --- | --- | --- | --- | --- | --- | --- | --- | --- |
|  |  |  |  |  |  | Lower | Upper |  |  |
| Climate anxiety | 0.00 | 0.05 | 0.00 | 0.07 | .946 | -0.10 | 0.10 | .001 | 6 |
| Loneliness | 0.31 | 0.08 | 0.20 | 3.78 | <.001 | 0.15 | 0.47 | .072 | 3 |
| Financial anxiety | 0.26 | 0.04 | 0.35 | 6.18 | <.001 | 0.18 | 0.34 | .153 | 1 |
| Health anxiety | 0.35 | 0.11 | 0.19 | 3.29 | .001 | 0.14 | 0.56 | .079 | 2 |
| COVID-19 worry | -0.00 | 0.05 | -0.00 | -0.02 | .987 | -0.09 | 0.09 | .007 | 4 |
| Age | 0.00 | 0.02 | 0.01 | 0.11 | .909 | -0.05 | 0.05 | .003 | 5 |
| Gender (Male) | 0.00 | 0.10 | 0.00 | 0.02 | .984 | -0.19 | 0.20 | .001 | 7 |
| Model fit: χ²(919) =2277.23, *p* <.001; CFI = 0.842, RMSEA = 0.058, SRMR = 0.058, *R*^2^ = 0.315. | | | | | | | | | |

*Note.* *N* = 447, Gender was coded as female = 0, male = 1. Predictor ranking is based on average contribution to the model’s *R*^2^ across all possible combinations of predictors.

**SF2 - Table S3**. SEM results of mental health regressed on emotion-based climate anxiety, with dominance analysis ranking (general population sample).

|  | B | SE_B_ | β | z | p | 95% CI of *B* | | *R*^2^ | Ranking |
| --- | --- | --- | --- | --- | --- | --- | --- | --- | --- |
|  |  |  |  |  |  | Lower | Upper |  |  |
| Climate anxiety | 0.07 | 0.05 | 0.06 | 1.32 | .188 | -0.04 | 0.18 | .006 | 7 |
| Loneliness | 0.72 | 0.10 | 0.41 | 7.44 | <.001 | 0.53 | 0.90 | .233 | 1 |
| Financial anxiety | 0.28 | 0.05 | 0.29 | 5.70 | <.001 | 0.19 | 0.38 | .156 | 2 |
| Health anxiety | 0.41 | 0.11 | 0.21 | 3.74 | <.001 | 0.19 | 0.62 | .136 | 3 |
| COVID-19 worry | 0.02 | 0.06 | 0.02 | 0.41 | .683 | -0.09 | 0.13 | .023 | 4 |
| Ukraine war worry | 0.05 | 0.04 | 0.05 | 1.24 | .216 | -0.03 | 0.13 | .008 | 6 |
| Age | -0.03 | 0.02 | -0.07 | -1.67 | .096 | -0.06 | 0.01 | .005 | 8 |
| Gender (Male) | -0.09 | 0.07 | -0.05 | -1.24 | .214 | -0.23 | 0.05 | .012 | 5 |
| Model fit: χ²(827) =2035.48, *p* <.001; CFI = 0.862, RMSEA = 0.062, SRMR = 0.055, *R*^2^ = 0.579. | | | | | | | | | |

*Note.* *N* = 385, Gender was coded as female = 0, male = 1. Predictor ranking is based on average contribution to the model’s *R*^2^ across all possible combinations of predictors.

**SF2 - Table S4**. SEM results of insomnia severity regressed on emotion-based climate anxiety, with dominance analysis ranking (general population sample).

|  | B | SE_B_ | β | z | p | 95% CI of *B* | | *R*^2^ | Ranking |
| --- | --- | --- | --- | --- | --- | --- | --- | --- | --- |
|  |  |  |  |  |  | Lower | Upper |  |  |
| Climate anxiety | 0.04 | 0.04 | 0.05 | 0.94 | .347 | -0.05 | 0.13 | .002 | 7 |
| Loneliness | 0.30 | 0.08 | 0.25 | 4.05 | <.001 | 0.16 | 0.45 | .099 | 2 |
| Financial anxiety | 0.20 | 0.04 | 0.30 | 4.65 | <.001 | 0.11 | 0.28 | .114 | 1 |
| Health anxiety | 0.09 | 0.09 | 0.07 | 1.05 | .294 | -0.08 | 0.26 | .057 | 3 |
| COVID-19 worry | 0.15 | 0.05 | 0.19 | 3.05 | .002 | 0.05 | 0.24 | .044 | 4 |
| Ukraine war worry | -0.02 | 0.03 | -0.03 | -0.66 | .512 | -0.08 | 0.04 | .003 | 6 |
| Age | -0.03 | 0.01 | -0.11 | -2.36 | .019 | -0.06 | -0.01 | .013 | 5 |
| Gender (Male) | 0.06 | 0.06 | 0.05 | 0.96 | .337 | -0.06 | 0.17 | .002 | 8 |
| Model fit: χ²(956) =2176.51, *p* <.001; CFI = 0.861, RMSEA = 0.058, SRMR = 0.057, *R*^2^ = 0.334. | | | | | | | | | |

*Note.* *N* = 385, Gender was coded as female = 0, male = 1. Predictor ranking is based on average contribution to the model’s *R*^2^ across all possible combinations of predictors.
