# Supplementary material for: Climate Anxiety in Perspective: A Look at Dominant Stressors in Youth Mental Health and Sleep
Source: Ann N Y Acad Sci. 2025 Sep 15;1552(1):249–62. doi: 10.1111/nyas.70057 (PMC12576874; doi:10.1111/nyas.70057)
Supplement: Supplementary file 3 — Supporting Material: nyas70057‐sup‐0003‐SuppFileS3.docx [file NYAS-1552-249-s002.docx]

**Supplemental File 3 – SEM regression of mental health and insomnia severity on emotion-based climate anxiety and other psychological stressors**

Emotion-based climate anxiety was measured using a scale adopted from Ogunbode et al. (2022). The scale is based on the state anxiety component of the State-Trait Anxiety Inventory (STAI: Spielberger, 1983). It measures the intensity of an individuals negative feelings regarding climate change at a specific point in time. In contrast to the Climate Change Anxiety Scale (CCAS), emotion-based climate anxiety does not assume that climate anxiety encompasses symptoms of psychological impairment or dysfunction. Below is the wording of the scale as presented to participants in this study:

**Emotion-based climate anxiety** (Ogunbode et al., 2022)

Take a moment to collect your thoughts and focus on how you feel about climate change. Please read each item below and use the rating scales provided to indicate how you feel about climate change at this present moment.

1. I feel calm (reversed)

2. I feel tense

3. I feel relaxed (reversed)

4. I feel anxious

5. I feel peaceful (reversed)

6. I feel worried

7. I feel terrified

Response (1) = Not at all, (2) = Somewhat, (3) = Moderately, (4) = Very much, (5) = Extremely.

The scale showed a good level of reliability across the student (α = .89) and general population samples (α = .90).

**SF3 - Table 1**. SEM results of mental health regressed on emotion-based climate anxiety, with dominance analysis ranking (student sample).

|  | B | SE_B_ | β | z | p | 95% CI of *B* | | *R*^2^ | Ranking |
| --- | --- | --- | --- | --- | --- | --- | --- | --- | --- |
|  |  |  |  |  |  | Lower | Upper |  |  |
| Climate anxiety | 0.01 | 0.06 | 0.01 | 0.19 | .848 | -0.10 | 0.13 | .001 | 7 |
| Loneliness | 0.79 | 0.10 | 0.41 | 7.61 | <.001 | 0.58 | 0.99 | .215 | 1 |
| Financial anxiety | 0.17 | 0.04 | 0.19 | 3.96 | <.001 | 0.09 | 0.26 | .081 | 3 |
| Health anxiety | 0.61 | 0.12 | 0.27 | 4.92 | <.001 | 0.37 | 0.86 | .131 | 2 |
| COVID-19 worry | -0.09 | 0.05 | -0.08 | -1.66 | .097 | -0.19 | 0.02 | .027 | 4 |
| Age | -0.03 | 0.03 | -0.04 | -1.08 | .280 | -0.09 | 0.03 | .001 | 6 |
| Gender (Male) | -0.12 | 0.11 | -0.04 | -1.06 | .289 | -0.34 | 0.10 | .005 | 5 |
| Model fit: χ²(793) =2119.01, *p* <.001; CFI = 0.845, RMSEA = 0.061, SRMR = 0.056, *R*^2^ = 0.462. | | | | | | | | | |

*Note.* *N* = 447, Gender was coded as female = 0, male = 1. Predictor ranking is based on average contribution to the model’s *R*^2^ across all possible combinations of predictors.

**SF3 - Table 2**. SEM results of insomnia severity regressed on emotion-based climate anxiety, with dominance analysis ranking (student sample).

|  | B | SE_B_ | β | z | p | 95% CI of *B* | | *R*^2^ | Ranking |
| --- | --- | --- | --- | --- | --- | --- | --- | --- | --- |
|  |  |  |  |  |  | Lower | Upper |  |  |
| Climate anxiety | 0.00 | 0.05 | 0.00 | 0.07 | .946 | -0.10 | 0.10 | .001 | 6 |
| Loneliness | 0.31 | 0.08 | 0.20 | 3.78 | <.001 | 0.15 | 0.47 | .072 | 3 |
| Financial anxiety | 0.26 | 0.04 | 0.35 | 6.18 | <.001 | 0.18 | 0.34 | .153 | 1 |
| Health anxiety | 0.35 | 0.11 | 0.19 | 3.29 | .001 | 0.14 | 0.56 | .079 | 2 |
| COVID-19 worry | -0.00 | 0.05 | -0.00 | -0.02 | .987 | -0.09 | 0.09 | .007 | 4 |
| Age | 0.00 | 0.02 | 0.01 | 0.11 | .909 | -0.05 | 0.05 | .003 | 5 |
| Gender (Male) | 0.00 | 0.10 | 0.00 | 0.02 | .984 | -0.19 | 0.20 | .001 | 7 |
| Model fit: χ²(919) =2277.23, *p* <.001; CFI = 0.842, RMSEA = 0.058, SRMR = 0.058, *R*^2^ = 0.315. | | | | | | | | | |

*Note.* *N* = 447, Gender was coded as female = 0, male = 1. Predictor ranking is based on average contribution to the model’s *R*^2^ across all possible combinations of predictors.

**SF3 - Table 3**. SEM results of mental health regressed on emotion-based climate anxiety, with dominance analysis ranking (general population sample).

|  | B | SE_B_ | β | z | p | 95% CI of *B* | | *R*^2^ | Ranking |
| --- | --- | --- | --- | --- | --- | --- | --- | --- | --- |
|  |  |  |  |  |  | Lower | Upper |  |  |
| Climate anxiety | 0.07 | 0.05 | 0.06 | 1.32 | .188 | -0.04 | 0.18 | .006 | 7 |
| Loneliness | 0.72 | 0.10 | 0.41 | 7.44 | <.001 | 0.53 | 0.90 | .233 | 1 |
| Financial anxiety | 0.28 | 0.05 | 0.29 | 5.70 | <.001 | 0.19 | 0.38 | .156 | 2 |
| Health anxiety | 0.41 | 0.11 | 0.21 | 3.74 | <.001 | 0.19 | 0.62 | .136 | 3 |
| COVID-19 worry | 0.02 | 0.06 | 0.02 | 0.41 | .683 | -0.09 | 0.13 | .023 | 4 |
| Ukraine war worry | 0.05 | 0.04 | 0.05 | 1.24 | .216 | -0.03 | 0.13 | .008 | 6 |
| Age | -0.03 | 0.02 | -0.07 | -1.67 | .096 | -0.06 | 0.01 | .005 | 8 |
| Gender (Male) | -0.09 | 0.07 | -0.05 | -1.24 | .214 | -0.23 | 0.05 | .012 | 5 |
| Model fit: χ²(827) =2035.48, *p* <.001; CFI = 0.862, RMSEA = 0.062, SRMR = 0.055, *R*^2^ = 0.579. | | | | | | | | | |

*Note.* *N* = 385, Gender was coded as female = 0, male = 1. Predictor ranking is based on average contribution to the model’s *R*^2^ across all possible combinations of predictors.

**SF3 - Table 4**. SEM results of insomnia severity regressed on emotion-based climate anxiety, with dominance analysis ranking (general population sample).

|  | B | SE_B_ | β | z | p | 95% CI of *B* | | *R*^2^ | Ranking |
| --- | --- | --- | --- | --- | --- | --- | --- | --- | --- |
|  |  |  |  |  |  | Lower | Upper |  |  |
| Climate anxiety | 0.04 | 0.04 | 0.05 | 0.94 | .347 | -0.05 | 0.13 | .002 | 7 |
| Loneliness | 0.30 | 0.08 | 0.25 | 4.05 | <.001 | 0.16 | 0.45 | .099 | 2 |
| Financial anxiety | 0.20 | 0.04 | 0.30 | 4.65 | <.001 | 0.11 | 0.28 | .114 | 1 |
| Health anxiety | 0.09 | 0.09 | 0.07 | 1.05 | .294 | -0.08 | 0.26 | .057 | 3 |
| COVID-19 worry | 0.15 | 0.05 | 0.19 | 3.05 | .002 | 0.05 | 0.24 | .044 | 4 |
| Ukraine war worry | -0.02 | 0.03 | -0.03 | -0.66 | .512 | -0.08 | 0.04 | .003 | 6 |
| Age | -0.03 | 0.01 | -0.11 | -2.36 | .019 | -0.06 | -0.01 | .013 | 5 |
| Gender (Male) | 0.06 | 0.06 | 0.05 | 0.96 | .337 | -0.06 | 0.17 | .002 | 8 |
| Model fit: χ²(956) =2176.51, *p* <.001; CFI = 0.861, RMSEA = 0.058, SRMR = 0.057, *R*^2^ = 0.334. | | | | | | | | | |

*Note.* *N* = 385, Gender was coded as female = 0, male = 1. Predictor ranking is based on average contribution to the model’s *R*^2^ across all possible combinations of predictors.
